# A systematic review and narrative synthesis of group self-management interventions for adults with epilepsy

**DOI:** 10.1186/s12883-017-0893-3

**Published:** 2017-06-17

**Authors:** Amelia Smith, Alison McKinlay, Gabriella Wojewodka, Leone Ridsdale

**Affiliations:** 1 GKT School of Medicine, King’s College London, London, SE1 1UL UK; 2 Institute of Psychiatry, Psychology & Neuroscience, Academic Neuroscience Centre, King’s College London, PO Box 57, London, SE5 8AF UK

**Keywords:** Self-management education, Epilepsy, Patient-education, Quality of life

## Abstract

**Background:**

Epilepsy is a serious and costly long-term condition that negatively affects quality of life, especially if seizures persist on medication. Studies show that people with epilepsy (PWE) want to learn more about the condition and some educational self-management courses have been trialled internationally. The objectives of this review were to evaluate research and summarise results on group self-management interventions for PWE.

**Methods:**

We searched Medline and PsycINFO for results published in English between 1995 and 2015. Only studies evaluating face-to-face, group interventions for adults with epilepsy were included. Heterogeneity in study outcomes prevented the carrying out of a meta-analysis; however, a Cochrane style review was undertaken.

**Results:**

We found eleven studies, nine of which were randomised controlled trials. There were variable standards of methodological reporting with some risk of bias. Seven of the studies used quality of life as an outcome, with four finding statistically significant improvements in mean total score. Two found an improvement in outcome subscales. One study included some additional semi-qualitative data.

**Conclusions:**

We identified promising trends in the trials reviewed. In particular, there were significant improvements in quality of life scales and seizure frequency in many of the interventions. However, considerable heterogeneity of interventions and outcomes made comparison between the studies difficult. Courses that included psychological interventions and others that had a high number of sessions showed more effect than short educational courses. Furthermore, the evidence was predominantly from pilot studies with small sample sizes and short follow-up duration. Further research is needed to better evaluate the role of group self-management interventions in outpatient epilepsy management.

## Background

Epilepsy is a long-term condition characterised by recurrent seizures, with a prevalence of around 1% in the general population [1, 2]. Common consequences of living with epilepsy include driving limitations, detrimental effects on education, unemployment, and diminished psychological wellbeing [3]. Stigma, frequency of seizures, and healthcare experiences also affect quality of life (QoL) in people with epilepsy (PWE) [4].

Epilepsy has significant financial and social costs. Direct costs are associated with a high rate of emergency admission that occurs with poorly-controlled epilepsy [5]. Emergency service use makes up the majority of admissions for epilepsy. Among all long-term conditions, epilepsy is the sixth most common cause of emergency admission in the United Kingdom [6, 7]. Reducing unnecessary emergency admissions is a key factor in helping to relieve financial pressure on healthcare services. Another major social issue is the indirect cost of epilepsy due to lost employment [8]. The health and social costs could be reduced and QoL improved via better outpatient management. However, around 40% of those diagnosed have poorly-controlled epilepsy and continue to have two or more seizures per year, [3] despite using antiepileptic drugs (AEDs). These figures highlight missed opportunities for epilepsy self-management.

Management of long-term conditions requires self-efficacy and empowerment, enabling patients to live as independently as possible and reducing the need to go to hospital [9]. For other long-term conditions, strategies for enabling such behaviour have been attempted within self-management education courses. A diabetes group intervention used in the United Kingdom, called DESMOND, is a cost-effective intervention, shown to improve biopsychosocial outcomes [10–12]. The programme is structured and can be run over one to two days for six hours in total [12]. Content is based on social learning theory [13] and is integrated into standard outpatient care for diabetes.

Early research evidence from North America [14] and Germany [15] suggested that group self-management courses had the potential to have a positive effect on health outcomes in PWE. The interest in group self-management for PWE has grown; however, the evidence base for developing self-management groups as standard outpatient care for PWE is still small. The objectives of this review were to evaluate recent research and summarise results of group self-management interventions for PWE. This was undertaken in the context that our group was conducting a trial of group self-management education intervention in the UK [16].

## Methods

### Study eligibility criteria

#### Population

PWE, adults aged 16 or over, without learning disabilities (due to the markedly different approaches required for educational programs in these populations [17]).

#### Intervention

Group self-management interventions were the focus of this review, irrespective of the study objectives (i.e., education, behavioural therapy, or a combination were included). We were interested in the psychological and social elements of face-to-face, group self-management courses. Studies using telemedicine were therefore excluded, as they are not provided face-to-face in groups.

#### Comparison

Treatment as usual or waitlist control.

#### Outcomes of interest

There was particular interest in QoL as this is the outcome favoured by the National Institute for Health and Care Excellence (NICE) [18, 19]. However, as there is no fixed consensus on the best measure for evaluating group interventions, we also included studies assessing other outcomes such as seizure frequency, psychological state, self-efficacy, and knowledge of epilepsy.

#### Exclusion criteria

Studies reporting trial protocol without results, one-to-one interventions, web- or telephone-based interventions, and samples including people with learning disabilities, children or non-epileptic seizures.

We searched for papers published from 1990 to 2015. A randomised controlled trial is considered the “gold standard” research design for evaluating the efficacy of an intervention; [20] however, we extended search criteria to include other forms of clinical trial (i.e., controlled outcomes design).

### Search strategy

We conducted electronic searches of the databases Medline and PsycINFO using the following keywords: epilepsy, seizures, self-care/self-efficacy, patient education/education programme, self-management, group intervention/complex intervention. A manual search of reference lists was performed to identify further relevant studies. For specific strategies for database searches refer to Appendix 1.

### Quality appraisal

All studies were assessed for quality using the CONSORT guidelines for reporting clinical trials [21]. Studies were assigned a number between 0 (description absent) and 2 (satisfactory description) according to how fully they described the four sections: trial design (methodology), participants (study sample and characteristics), interventions and study outcomes. Appraisal was carried out by two independent assessors and studies were discussed as a group to resolve any disagreements. The Cochrane approach was used to categorise the studies into ‘high’, ‘low’ or ‘unclear’ risk of bias with regard to random sequence generation and allocation concealment [22]. As it is not possible to double-blind a group self-management intervention, this was not included in the quality assessment.

## Results

### Study selection

**Fig. 1:**
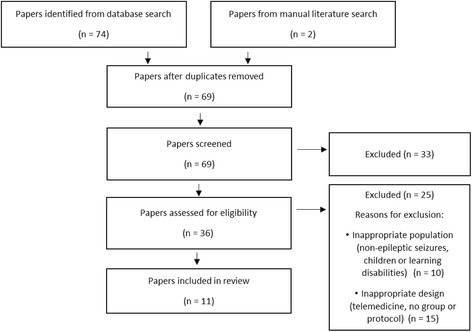
Search tree

The first author conducted the initial literature search, which was repeated by the second author. The initial Medline search resulted in 42 papers being identified (Fig. 1). After examining the titles and abstracts, nine were unrelated and were excluded. Five studies on non-epileptic seizures (psychogenic seizures and febrile convulsions) were excluded also [23–27]. Five more were excluded as their participant population consisted of children (under 16) or patients with learning disabilities. Of the remaining papers, six were identified as involving a telephone or web-based intervention and were also excluded. Finally, some studies were excluded because no group intervention was administered. The papers described either individual care programmes or trial protocols with no results.

PsycINFO returned 32 results; three new papers were identified, but none were eligible for inclusion (two protocols, one telemedicine). Two studies were included from the manual literature search, resulting in 11 studies in total.

### Search results

Of the 11 studies included with this review (Table 1), nine were described by their authors as randomised controlled trials [15, 28–30, 31, 32–35] and the remaining two as controlled outcome designs [36, 37]. The first study included a matching strategy for allocating participants to control or treatment group [37]. The second study used random assignment but did not provide more specific protocol information [36]. After synthesising review results, we established that due to outcome heterogeneity a meta-analysis would not be possible.

**Table 1:** Summary of included papers

Author	Participants	Intervention (int.)	Facilitator	Control	Outcome measures	Main findings
Aliasgharpour et al. [33]	60 PWE randomised Aged 18–70 Diagnosed for ≥1 yr. Experienced seizures in the past year	1 month educational int. Four 2 hr sessions Face-to-face lectures with case histories, demonstrations and information leaflets to take away Group size: 4–6. Discrete intervention	Master’s student in nursing	TAU	ESMS Pre-intervention and 1 month follow-up	Self-management score significantly improved in int. vs control
Au et al. [37]	17 adults with epilepsy (age range not specified) ≥ 2 seizures a month	8 week psychological CBT int. Eight 2 hr structured sessions Group size: 8 Discrete intervention	2 clinical psychologists trained in seizure management	TAU	QOLIE-31, ESES, seizure frequency 3 months pre-int. and 3 month follow-up	Significant improvement in QoL and self-efficacy scores in intervention group vs control No difference in seizure frequency
Fraser et al. [35]	83 PWE randomised Age ≥ 18 Diagnosed for ≥6 months	8 week psychoeducational int. Eight 75 min sessions Presentations, facilitated discussion and workbook Group size: 6–8. Discrete int.	Rehabilitation psychologist and trained peer mentor	WC	QOLIE-31, ESMS, ESES, PHQ-9, GAD-7 Pre-int., 8 week and 6 month follow-up	QoL, self-efficacy and PHQ-9 scores improved at 8 weeks in int. group but not significant at 6 months. Self-management significantly improved at 6 months
Helde et al. [29]	111 PWE aged 16–70 Diagnosed for ≥1 yr. ≥ 1 seizure in the past yr	1 day educational int. Information and discussion about epilepsy and psychosocial aspects Group size: 5–11 Additional input: telephone follow-up and 1–1 counselling from nurse	MDT (the study nurse, neurologist, social worker, and neurophysiologist)	TAU	QOLIE-89 and general patient satisfaction score Pre-randomisation and at 2 year follow-up	Significant improvement in QoL score in int. group at follow-up but no sig difference between int. and control Significant increase in satisfaction in int. vs control
Ibinda et al. [34]	738 PWE randomised 581 data analysed ≥ 1 seizure in the past yr	1 day educational int. Information about epilepsy provided using role play, discussion, narratives and brochure on the topics was given Group size: up to 20. Discrete int.	Non-specific ‘researchers and field staff’	WC	AED adherence, seizure frequency, KEBAS (Kilifi epilepsy beliefs and Attitudes scores) Pre-int. and after 1 year follow-up	Significantly improved KEBAS scores in int. vs control at follow-up. No difference in adherence or seizure frequency between the groups (both improved significantly)
Losada-Comacho et al. [28]	182 women with epilepsy randomised Age ≥ 18 Diagnosed ≥1 yr. ≥ 1 seizure in the past 3 yrs	Educational int. part of pharmaceutical care programme Monthly lectures and information brochures Group size: not specified Additional input: 1–1 medication reviews. Given adherence aids and seizure journals	Pharmacist trained in epilepsy management	TAU and seizure Information brochure	QOLIE-31, seizure frequency, adverse events,CES-D (depression), Haynes-Sackett and Morinski-Green tests (medication adherence) Pre-int. and after 6 month follow-up	Highly significant improvement in QoL score between int. and control groups at follow-up. Other outcomes not reported in the paper
Lundgren et al. [30]	27 PWE randomised Aged 21–55 ≥ 4 seizures in the past 3 months	Psychological ACT int. Two 3 hr sessions Group size: 6–8 Additional input: Two individual 90 min sessions, individualised seizure control techniques	2 clinical psychologists	Supportive therapy	WHOQOL-BREF, SWLS, seizure index Pre-int, post-int, and at 6 month and 1 year follow-up	Significant improvement in seizure index at all time points post-int. in int. group vs control. Significant improvement in QoL scores in int. group after 1 year
May and Pfafflin, [15]	383 PWE randomised Age ≥ 16 Any duration or severity of epilepsy	2 day educational int. Interactive course with 9 modules aiming to improve knowledge of epilepsy and psychosocial factors Group size: not specified. Discrete int.	Non-specific ‘trainers’	TAU	SF-36, Depression Scale D-S′, Rosenberg self-esteem, stigma, restrictions due to epilepsy, epilepsy-related fears and mobility and leisure scales, specifically developed epilepsy knowledge and coping with epilepsy scales. Seizure frequency Pre-int. and 6 month follow-up	Significant improvement in knowledge and coping scales (specifically developed) in int. group. Significant improvement in seizure frequency in int. group. No impact on QoL (SF-36 score)
McLaughlin and McFarland, [32]	37 older adults with epilepsy Age ≥ 60	6 week psychological CBT int. Six 2 h sessions Spector et al. protocol, modified for older adults with less content and diaries to aid memory Group size: 6–7. Discrete int.	Psychologist with epilepsy expertise	Relaxation training	GDS, CIDI-auto, WPSI, seizure frequency Pre-int, post-int. and after 3 month follow-up	Significant improvement in seizure frequency in int. group vs control. No significant difference between groups in other measures but depression and psychosocial functioning improved in both.
Olley et al. [36]	30 PWE allocated to groups Aged 21–65	2 day psychoeducational int. Educational sessions and group discussion on epilepsy, stigma & management Group size: not specified. Discrete int.	Non-specific ‘researcher/therapist’	WC	CCEI, BDI (psychological symptoms), knowledge about epilepsy Pre-int, post-int and after 2 month follow-up	Significant improvement in int. group vs control in psychological scales and increased knowledge about epilepsy at follow-up
Pramuka et al. [31]	55 PWE randomised Age ≥ 18	6 week psychoeducational int. Six 2 hr sessions Presentations, activities and discussion on medical and self-management topics Written information provided Group size: 4–12. Discrete int.	2 ﻿psychologists and 1 research associate Guest lecture by nurse specialist	TAU	QOLIE-89, ESES, WPSI (psycho-social factors), locus of control scale, MCMI-III (depression) Pre-int. and 1 month follow-up	Trends in improved direction in all measures, but only one QoL subscale showed significant improvement in int. group vs control at follow-up.

*PWE* people with epilepsy; *yr.* year; *int* intervention; *hr.* hour; *TAU* treatment as usual; *ESMS* Epilepsy Self-Management Scale; *CBT* Cognitive Behaviour Therapy; *QOLIE* Quality of Life in Epilepsy; *ESES* Epilepsy Self-Efficacy Scale; *QoL* Quality of Life; *WC*waitlist control; *PHQ*-*9* Patient Health Questionnaire-9; *GAD*-*7* Generalised Anxiety Disorder-7; *MDT* multidisciplinary team; *ACT* Acceptance and Commitment Therapy; *WHOQOL*-*BREF* World Health Organisation quality of life – abbreviated version; *SWLS*: Satisfaction with Life Scale; *SF*-*36* 36-item short form survey; *GDS* Geriatric Depression Scale; *CIDI*-*auto* Composite International Diagnostic Interview; *WPSI* Washington Psychosocial Seizure Inventory; *CCEI* Crown-Crisp Experiential Index; *BDI* Beck Depression Inventory; *MCMI*-*III*: Millon Clinical Multiaxial Inventory -III

All studies focused on the effects of a group intervention in PWE, with one exclusively recruiting women [28] and another looking at older adults [32]. Most studies targeted poorly-controlled epilepsy; however, the definition of ‘poorly-controlled’ varied and four studies did not specify a minimum seizure frequency.

We assessed 11 studies for quality and risk of bias (Table 2). For most studies, the participants and outcome measures were well described. Trial design was the least well described throughout the 11 studies followed by the intervention description. Eight of the 11 studies had low risk of bias for the generation of the sequence allocating participants to treatment groups. In most study designs for self-management courses, the participants and facilitators will be un-blinded. Other research staff, such as those assessing participants and analysing data should have remained blinded to minimise bias. The concealment of group allocation was not described in the majority of studies.

**Table 2:** Quality ratings and risk of bias

Study	Quality appraisal					Risk of bias appraisal			
	Trial design	Participants	Intervention	Outcomes	Total (max 8)	Sequence generation	Risk of bias	Allocation concealment	Risk of bias
Aliasgharpour et al. [33]	1	1.75	2	2	6.75	Random number table	Low	Not described	Unclear
Au et al. [37]	0.75	2	2	2	6.75	Matched design	Unclear	Not described	Unclear
Fraser et al. [35]	1.75	2	1.75	2	7.5	Random number generator	Low	Not described	Unclear
Helde et al. [29]	1.25	2	2	2	7.25	Computer-generated block randomisation	Low	Research assistant blinded	Low
Ibinda et al. [34]	1.25	1.25	0.5	1.5	4.5	Computer-generated randomisation	Low	Not described	Unclear
Losada-Camacho et al. [28]	2	2	1.75	2	7.75	Drawing of ballot papers	Low	Sequentially numbered, opaque, sealed envelopes	Low
Lundgren et al. [30]	1	2	2	2	7	Computer-generated randomisation	Low	Not described	Unclear
May and Pfafflin. [15]	1.25	2	0.75	1.5	5.5	Not described	Unclear	Not described	Unclear
McLaughlin and McFarland [32]	1	1.75	2	2	6.75	Computer-generated randomisation	Low	Not described	Unclear
Olley et al. [36]	1.5	2	1.25	1.75	6.5	Alternate clinic visit	High	Not described	Unclear
Pramuka et al. [31]	1.5	2	2	2	7.5	Random number table	Low	Consecutively numbered, sealed envelopes	Low

### Facilitators and treatment groups

Interventions were categorised as educational, [15, 28, 29, 33, 34] psychological (e.g. behavioural therapy) [30, 32, 37] and psychoeducational programmes (i.e. both) [31, 35]. Psychological and psychoeducational interventions were delivered by psychologists, whereas educational sessions were delivered by a range of practitioners. In two studies, [34, 36] instructors’ qualifications were not clearly specified (e.g. ‘researcher’ or ‘staff’). In one study, [15] facilitators were specified in a previous publication to be physicians, nurses, psychologists, social workers and occupational therapists.

The treatment of the control group varied across the studies. In six of the studies, [15, 28, 29, 31, 37, 33] controls received treatment as usual (TAU). Additional input was given to the controls alongside TAU in one study [33] in the form of two short telephone calls. In two, [30, 32] a control for attention was offered, for example, supportive therapy, involving equivalent attention from professionals, but without any active teaching or advice. Three studies [34–36] used a waitlist control design and offered intervention materials after follow-up.

### Outcomes

The most frequently assessed outcome measure was QoL, used in seven studies included within this review [15, 28–30, 31, 35, 37]. The Quality of Life in Epilepsy questionnaire (QOLIE-31/QOLIE-89) [38, 39] was commonly chosen to assess it [28, 29, 31, 35, 37]. Six studies [15, 28, 30, 32, 34, 37] measured seizure frequency as a marker of control and three assessed self-efficacy, [31, 35, 37] using the Epilepsy Self-Efficacy Scale (ESES) [40]. Two studies [33, 35] used a specific self-management outcome measure called the Epilepsy Self-Management Scale (ESMS) [41]. Different measures were used to assess psychological symptoms, such as the Patient Health Questionnaire (PHQ-9) [42]. Comparison between the studies was complicated, due to the heterogeneity of outcome measures that were used.

### Follow-up

The duration of follow-up ranged from one month [33] to two years [29] with a median follow-up period of six months. All studies recorded outcome measures at baseline before the intervention and at the end of follow-up. Four also took further measures immediately post-intervention or halfway through the follow-up period [30, 32, 35, 36].

## Overview of findings

### Quality of life

Of the seven studies using QoL outcome measures, three showed no significant difference in total QoL scores between the intervention and control groups [15, 29, 31]. Fraser et al. showed a significant improvement in QOLIE-31 scores eight weeks post-intervention, but no statistical significance between means after six months [35].

Two psychological interventions showed an improvement in QoL scores at follow-up. The cognitive behavioural therapy study demonstrated a significant QOLIE-31 score improvement in the intervention group compared with the control at the end of a three month follow-up [37]. Emotional Wellbeing was the only subscale that was significantly increased. In the trial by Lundgren et al [30]. there was a significant improvement in WHOQOL-BREF score (World Health Organisation Quality of Life Assessment abbreviated version) [43] in the acceptance and commitment therapy group, compared to supportive therapy at the final one year follow-up. The Satisfaction with Life Scale (SWLS) [44] score was improved at all three time points after the intervention.

One education-focused study showed an improvement in QoL score. The trial by Losada-Camacho et al. found significant improvement in QOLIE-31 after six months [28]. The intervention package was offered to women only, and focused on pharmaceutical aspects of epilepsy self-management. As one of the co-interventions, participants received advice and monitoring at set intervals from a pharmacist. At six-month follow-up, the total score increased in the intervention group by 12.45 points compared with a 2.61 increase in the controls.

### Seizure frequency

Six studies measured seizure frequency. Three found that seizure frequency decreased significantly in the intervention group compared with controls [28, 30, 32]. However, two studies did not find a significant difference in seizure frequency post-intervention. Au et al. described a small improvement in the intervention group but this was not significant, [37] whereas, Ibinda et al. found a decrease in both groups but no difference between them [34]. One study did not report seizure frequency despite collecting this data [28]. The reason for this was unclear; however, it may be due to poor compliance with seizure diaries as only 37.5% of participants returned them.

### Self-management and self-efficacy

Two studies using the ESMS showed promising findings after group intervention delivery. Aliasgharpour et al. found a significant difference between the groups post-intervention with an increase in self-management score in the intervention group but none in the control [33]. Likewise, Fraser et al. found a treatment effect favouring the intervention at eight weeks which, although small, remained significant at the six month follow-up [35].

Three studies used the ESES to measure self-efficacy [31, 35, 37]. Fraser et al. found a significant treatment effect in ESES score after eight weeks but this did not persist at six months. Au et al. found a significant improvement in score in the intervention group compared to control. Findings from Pramuka et al. showed a trend towards improvement in the intervention group but this was not significant.

### Psychological symptoms

Two of the six studies which used symptom scales indicating psychological comorbidity showed statistically significant improvement [35, 36]. Fraser et al. found a statistical significant change in PHQ-9 [39] score with reductions in depressive symptom severity at eight weeks post-intervention; however, the difference between intervention and control was not statistically significant at six months. Olley et al. found an improvement in psychological state compared with controls but, with only two months follow-up, no long-term benefit was demonstrated [36]. The others found improvements which were not significant [28, 31, 32]. In the study by Losada-Camacho et al. the psychological outcome results were not reported, [28] nor were they published elsewhere.

## Discussion

Quality assessment revealed gaps in the methodological reporting of many studies, especially regarding trial design. Risk of bias was assessed and most papers fell into the ‘unclear’ category due to insufficient description of treatment concealment. We paid particular attention to who delivered each intervention and how thoroughly this was described. Information about the role and training of facilitators is needed in order to replicate an intervention and apply it to other healthcare settings.

The usefulness of study findings is somewhat diminished in those that lacked thorough reporting of treatment fidelity. Most interventions were delivered multiple times, sometimes by different facilitators; however, only one study discussed means of ensuring treatment fidelity but the results were not presented [30]. Group interventions may not be delivered consistently and without reporting on measures to prevent deviation, it is uncertain whether interventions were delivered as planned.

All studies reported significant improvement in the intervention group compared with controls in at least one measure. More than half of the studies assessing QoL found some positive effect from the intervention but the duration of that effect is varied. Five studies repeated the QoL measures at least six months after the intervention and only two found a sustained significant improvement [28, 30]. These both consisted of sessions which were spread out over time, which may be a better intervention strategy than one-off courses. Fraser et al. also delivered their intervention over a period of 8 weeks but were unable to demonstrate a significant effect at 6 month follow-up [35]. They suggested the addition of a booster course to the programme. It is likely that other factors may also influence the duration of improvement.

It was difficult to identify mechanisms that make a group intervention for PWE effective. This is particularly true of co-interventions, where group effects cannot be separated out from additional support. Psychological interventions performed well on QoL and seizure frequency (Table 1) [36, 37, 32] although they had very small sample sizes (*n* = 17, 27 and 37). This suggests that including behavioural therapy in self-management interventions may be important to affect QoL.

Although some studies found positive trends for QoL improvements from baseline measurements, the scores were not necessarily statistically different between study groups. Many were pilot studies with small sample sizes and may not have had adequate power to detect important changes. One study that encountered this problem calculated a required sample size of 180 participants but recruitment difficulties resulted in a final sample of only 55 [31]. Larger-scale trials are needed to explore this further.

Another factor that may have contributed to the lack of significant effect is the use of unsuitable outcomes. Two studies found improvements only in measures developed specifically for the trial, despite including a variety of other outcomes [15, 34]. This suggests that the existing scales may not be optimal for evaluating effects of self-management interventions in this setting.

The diversity of outcome measures used across all studies made comparison difficult. There were five scales used to assess QoL, of which two were epilepsy-specific. Four studies did not use any QoL questionnaires although their chosen outcomes (e.g. seizure frequency) can be assumed to affect QoL. This highlights lack of consensus on the most appropriate measures for complex interventions in epilepsy.

Only one study used semi-qualitative data in their evaluation, collecting written comments from participants in an open-ended satisfaction survey [35]. They received positive comments about the intervention with 47% of respondents mentioning the value of meeting other PWE. However, response rates were uncertain and there were no interviews to explore participants’ views. Guidelines from the Medical Research Council recommend the inclusion of qualitative methods as part of a process evaluation for complex interventions [45]. In light of this, it would be useful to collect further qualitative data on how and why group self-management interventions ﻿are beneficial. 

The diversity of study settings also complicates interpretation. As trials were conducted across 13 countries with wide-ranging cultural and socioeconomic backgrounds, baseline standards of care and health literacy were likely different. Furthermore, participants in each setting may not be representative of the wider population. This was particularly relevant in the Seattle-based study which typically recruited highly educated participants, some of whom were volunteers, limiting the generalisability of their findings to other western countries in which health care is provided to all socio-economic groups [35].

There are several limitations associated with the present review. We were unable to perform a meta-analysis due to significant heterogeneity of outcome measures observed across the studies. Moreover, not all Cochrane review tools were utilised when evaluating the quality of studies included. Findings from the review may also be limited due to biases arising from initial search criteria (i.e., article language and publication date).

### Suggestions for future research

Based on the evidence available to date, it seems that QoL is rarely affected long term by educational interventions. Thus to have a better chance of affecting QoL, self-management interventions should include psychological components. If group self-management education is to be offered as part of standard outpatient care for PWE, as DESMOND is for people with diabetes, then future research should examine the feasibility and cost-effectiveness of implementation. Although this review did not specifically search for published articles with health economics data, we found no discussion of the cost of implementing self-management education courses for PWE in the community. This would be valuable in the context of similar programmes being implemented in countries with public healthcare systems.

## Conclusions

The studies evaluating group self-management interventions for PWE found encouraging results. There is some evidence that psychoeducational measures can be delivered to improve self-management, seizure control, and QoL in adults with poorly-controlled epilepsy. The MOSES programme has been offered in German-speaking countries for over a decade and other countries are investigating similar interventions [15]. Promising findings, along with demand from patients for more information about epilepsy, are indications for continued interest in group self-management interventions for PWE.

This review illustrates the need for clarity regarding outcome measures in this field of epilepsy research. Additionally, large-scale trials of group self-management interventions, combining quantitative, qualitative, and cost-effectiveness data, are required in the future.

## Appendix 1

Search strategies

### Medline (Ovid) search strategy

1. exp. Epilepsy/
2. epilep*
3. seizures
4. exp. Self Care/
5. exp. Self Efficacy/
6. exp. Patient Education as Topic/
7. educational program
8. self-management
9. group intervention
10. complex intervention
11. 1 or 2 or 3
12. Limit 11 to (English language and yr. = “1995 - Current”)
13. 4 or 5 or 6 or 7 or 8 or 9 or 10
14. Limit 13 to (English language and yr. = “1995 - Current”)
15. 12 and 14
16. Limit 15 to (clinical trial or controlled clinical trial or pragmatic clinical trial or randomized controlled trial)

### PsycINFO search strategy

1. Exp Epilepsy/
2. epilep*
3. seizures
4. 1 or 2 or 3
5. self-management
6. exp. Self Efficacy/
7. group intervention
8. complex intervention
9. patient education
10. self care
11. educational program*
12. 5 or 6 or 7 or 8 or 9 or 10 or 11
13. 12 and 4
14. limit 13 to (English language and yr. = “1995-Current”)

## Abbreviations

DESMOND: Diabetes education and self-management for ongoing and newly diagnosed
ESES: Epilepsy self-efficacy scale
ESMS: Epilepsy self-management scale
MOSES: Modular service package epilepsy
PHQ-9: Patient health questionnaire 9
PWE: People with epilepsy
QoL: Quality of life
QOLIE: Quality of life in epilepsy
SWLS: Satisfaction with life scale
TAU: Treatment as usual
WHOQOL-BREF: World health organisation quality of life abbreviated version
